# Long-term motor skill training with individually adjusted progressive difficulty enhances learning and promotes corticospinal plasticity

**DOI:** 10.1038/s41598-020-72139-8

**Published:** 2020-09-24

**Authors:** Lasse Christiansen, Malte Nejst Larsen, Mads Just Madsen, Michael James Grey, Jens Bo Nielsen, Jesper Lundbye-Jensen

**Affiliations:** 1 Department of Nutrition Exercise and Sports, University of Copenhagen, Copenhagen, Denmark; 2 Department of Neuroscience, University of Copenhagen, Copenhagen, Denmark; 3 Danish Research Centre for Magnetic Resonance, Centre for Functional and Diagnostic Imaging and Research, Copenhagen University Hospital, Amager and Hvidovre, Hvidovre, Denmark; 4 School of Health Sciences, Faculty of Medicine and Health Sciences, University of East Anglia, Norwich Research Park, Norwich, United Kingdom; 5 Department of Sports Science and Clinical Biomechanics, University of Southern Denmark, Odense, Denmark

**Keywords:** Long-term memory, Motor cortex, Neural circuits, Neurophysiology

## Abstract

Motor skill acquisition depends on central nervous plasticity. However, behavioural determinants leading to long lasting corticospinal plasticity and motor expertise remain unexplored. Here we investigate behavioural and electrophysiological effects of individually tailored progressive practice during long-term motor skill training. Two groups of participants practiced a visuomotor task requiring precise control of the right digiti minimi for 6 weeks. One group trained with constant task difficulty, while the other group trained with progressively increasing task difficulty, i.e. continuously adjusted to their individual skill level. Compared to constant practice, progressive practice resulted in a two-fold greater performance at an advanced task level and associated increases in corticospinal excitability. Differences were maintained 8 days later, whereas both groups demonstrated equal retention 14 months later. We demonstrate that progressive practice enhances motor skill learning and promotes corticospinal plasticity. These findings underline the importance of continuously challenging patients and athletes to promote neural plasticity, skilled performance, and recovery.

## Introduction

Throughout our lifespan, numerous motor skills have to be acquired and retained in memory. Behavioural plasticity, as observed in motor skill learning, is contingent on underlying adaptations in the central nervous system, i.e. mechanisms of neuroplasticity. In humans, neuroplasticity is commonly investigated indirectly e.g. using brain stimulation and neuroimaging techniques (see^1^ for review of early, seminal papers). The corticospinal tract plays a key role in motor control^2^, and plastic changes in the corticospinal pathway are important mechanisms for the acquisition of skilled movement^3^. In humans, the early phase of motor skill acquisition is accompanied by a transient increase in corticospinal excitability (CSE). This is evident as an increase in the size of responses to transcranial magnetic stimulation over the representation of the trained muscle following a single session of motor skill training^4–6^. This initial increase in CSE can increase further with multiple days and weeks of motor training, but it eventually stagnates and decreases as training progresses further without additional challenge^7^. In contrast to this observation, studies of expert musicians and athletes have found larger cortical representation and increased CSE of their trained muscles and movements suggesting that continued training is accompanied by persistent corticospinal plastic changes^8–10^. Such long-term plastic changes in the human corticospinal pathway accompanying motor training are not well understood, and the role of behavioural determinants in particular has only been studied sparsely^7,11–13^.

In contrast, plastic changes in the primary motor cortex (M1) and the corticospinal pathway following weeks of practice have been extensively reported in murine models of motor skill learning^14–21^ and M1 has been shown to be a pivotal locus for neuronal changes underlying both associative^22–24^, sequence^25^ and motor skill^25–27^ learning in primates. Furthermore, evidence from rodents^28,29^ and primates^26^ indicates that learning per se rather than use drives long-term plastic changes. In support of this, previous work in humans has shown that training-induced increases in corticospinal excitability reach a plateau and may even decline after weeks of training^7^. However, the decline in CSE is not associated with a corresponding decrease in performance. This suggests that increased corticospinal excitability is more likely related to the acquisition of new motor skills and does not relate directly to performance in the trained task. In contrast to the longitudinal studies, cross-sectional studies have reported that expert musicians, proficient Braille readers and athletes have larger sensorimotor cortical representation of their trained hand compared to non-experts or their own non-trained hand^8,9,30–33^.

One possible explanation for these seemingly contrasting findings may be that training for elite athletes and expert musicians is not characterized by practicing discrete laboratory tasks but rather by *deliberate practice* in which their skills are developed and expanded through progressive challenge^34^. The continuous progression in task demands and the tailoring of demands to individual levels of motor proficiency likely augments plastic changes in the corticospinal system, resulting in superior motor performance compared to conventional non-progressive motor practice.

This could explain the differences in cortical representation and CSE that have been observed between novices and experts^9,31^. In support of this hypothesis, we recently demonstrated that 4 days of training a visuomotor accuracy task with progressively adjusted task difficulty resulted in superior learning accompanied by transient increases in CSE after both the first and last practice session^4^. In contrast, maintaining task difficulty at the baseline level was only accompanied by an increase in CSE on the first day of practice. This confirms earlier results suggesting that distinct neurophysiological processes are involved in early versus later stages of learning^35^. Altogether, these results suggest that transient within-session changes in CSE are related to top-down processes involved in skill learning whilst automaticity is low.

Here we aim to extend the our previous findings^4^ by testing the effects of long-term progressive motor training on corticospinal plasticity and skill acquisition. We hypothesize that individually tailored progressive training (PT) that continuously challenges and engages the learner will potentiate the changes in CSE accompanying nonprogressive training (NTP) and resemble those previously found in cross-sectional studies of virtuosi and elite athletes. Furthermore, we aim to provide *proof-of-principle* that progressive practice promotes learning. Finally, we explore the effects of PT on long-term retention of skill and its potential relation to corticospinal plasticity.

## Results

Group means for motor performance and electrophysiological measures are presented in Tables 1, 2 and 3. Participants were matched based on their ‘Baseline’ motor performance and randomly allocated to the two training groups, i.e. progressive training (PT) or nonprogressive training (NPT) to ensure similar ‘Baseline’ motor performance between groups. It should be noted that the task difficulty was identical for all participants during the first training session, i.e. on Day 1.

**Table 1:** Motor performance scores for the progressive and non-progressive training groups.

Group	N	Task level	Day 1	6 weeks	Retention 1
PT	12	Day 1 level	9.9 ± 1.1	19.2 ± 1	20.4 ± 1.3
NPT	12		9.7 ± 1.0	19.7 ± 1.6	20.4 ± 0.8
PT	6	End level	NT	9.6 ± 0.9	10.3 ± 0.8
NPT	6		NT	4.6 ± 0.5	5.1 ± 0.6

Motor performance scores on ‘Day 1’ and ‘End’ task level are reported from before training, after 6 weeks of training and for the retention test 8 days after end of the training protocol. The scores are non-normalized group mean values ± s.e.m.

*NT* not tested.

**Table 2:** Motor performance for the subset of participants tested in Retention test 2—after 14 months.

Group	N	Task level	6 weeks	Retention 1	Retention 2
PT	7	Day 1 level	20.3 ± 2.5	21.8 ± 5.7	17.3 ± 2.6
NPT	7		18.6 ± 4.3	20.3 ± 3.2	16.6 ± 5.1
PT	5	End level	8.4 ± 1.4	10.4 ± 3.2	4.4 ± 1.0
NPT	5		4.8 ± 1.3	4.4 ± 1.9	3.8 ± 1.4

Motor performance scores on ‘Day 1’ and ‘End’ task difficulty are reported for the first day of training, after 6 weeks of training, and for the retention test 1 and 2, 8 days and 14 months after end of the training protocol. The scores are non-normalized group mean values ± s.e.m.

**Table 3:** Electrophysiological parameters for the progressive and non-progressive training groups.

Group	N	Parameter	Day 1	2 weeks	4 weeks	6 weeks	Retention 1
PT	12	AURC % of Mmax × % of rMT	884 ± 105	1,755 ± 279	1,695 ± 261	1,449 ± 249	1,908 ± 303
NPT	12		1,425 ± 292	1,396 ± 239	1,330 ± 311	1,454 ± 322	1,476 ± 254
PT	12	rMT % of max stimulator output	38.3 ± 1.3	34.8 ± 1.2	34.7 ± 1.4	34.3 ± 1.4	35.7 ± 1.9
NPT	12		40.8 ± 1.7	38.3 ± 1.6	38.8 ± 1.7	37.7 ± 1.7	38.3 ± 1.6
PT	12	MEPmax % of M_max_	16.8 ± 2.2	27.9 ± 5.0	29.1 ± 4.6	25.1 ± 5.0	29.7 ± 3.9
NPT	12		26.9 ± 4.7	26.6 ± 3.6	25.5 ± 5.7	25.4 ± 4.6	26.5 ± 3.7
PT	12	I50 TMS intensity (% of rMT)	124.8 ± 3.3	115.4 ± 2.8	118.8 ± 2.4	117.3 ± 4.1	115.9 ± 5.1
NPT	12		128.9 ± 3.4	128.9 ± 5.3	122 ± 6.6	121.2 ± 4.1	125.5 ± 4.8
PT	12	Slope % of Mmax/% of rMT	11.7 ± 0.9	12.9 ± 1.9	15.3 ± 2.1	12.8 ± 1.7	12.4 ± 1.5
NPT	12		14.5 ± 1.6	15.2 ± 1.8	13.5 ± 1.8	13.9 ± 2.0	15.0 ± 2.2

Electrophysiological parameters: resting Motor Threshold (percentage of maximal stimulator output) and MEP_max_ (percentage of M_max_), I50 and slope values for the progressive and non-progressive training group at Baseline, after 2, 4 and 6 weeks of training and for retention test 1. The scores are non-normalized group mean values ± s.e.m.

### Changes in motor performance during the first day of training

**Figure 1:**
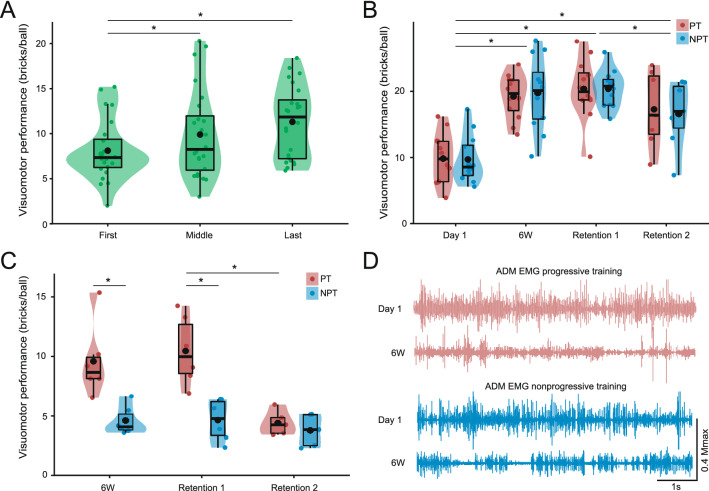
Motor performance and EMG activity during training. (**A**) Box- and density plots of performance from the first (left), middle (middle) and last (right) 4 min of the first training session. Each data point (green dot) represents the accumulated score from 4 min of training from one participant. Asterisk denotes a significant effect of time (p < 0.05, n = 24). (**B**) Box- and density plots of long-term comparisons of performance on the ‘Day 1’ task level for both groups. Asterisks denote significant difference from baseline performance and from Retention 1 to 2 across the two groups (p < 0.05, n = 24). (**C**) Box- and density plots of motor performance at ‘End’ task Level (n = 12). Asterisks denote a significant difference between the two groups (p < 0.05) and from Retention 1 to 2 within the PT group. (**A**–**C**) Coloured dots represent individual data. Whiskers represent highest and lowest value within 1.5 inter quartile range. Bold horizontal lines signify median values, the lower and upper hinges correspond to the first and third quartiles, and the means are represented by black dots. Please note that the scaling of Y-axes differs between the three violin plots. (**D**) ADM EMG activity, normalized to M_max_, during the first and last training session for one participant from each group. No statistical comparisons were made for EMG activity obtained during training.

Motor performance during the initial training session was assessed during the first, middle and last 4 min of training. Likelihood ratio tests of the mixed effects model showed that there was neither an interaction between GROUP and TIME (Chi^2^(2) = 0.44, p = 0.8) nor a main effect of GROUP (Chi^2^(1) = 0.01, p = 0.91). There was a robust effect of TIME (Chi^2^(2) = 18.1, p < 0.001), and post-hoc Tukeys test showed that motor performance was significantly higher in the middle (9.9 ± 1.5) and last (11.6 ± 1.2) compared to the first 4 min of training (8.3 ± 1) (z = 2.62, p = 0.026 and z = 4.68, p < 0.001). There was no significant difference between the middle and last training block (z = 2.06, p = 0.12) . Single subject and group data from the first training session is illustrated in Fig. 1A.

### Long-term changes in motor performance at the ‘Day 1’ task level

The longitudinal effect of training was assessed as motor performance after 6 weeks of practice and reassessed after 8 days of detraining (retention test 1) and again 14 months later in a subsample of participants (retention test 2). Motor performance at these time points was assessed at the same level of task difficulty as ‘Day 1’ and compared to ‘Day 1’ motor performance taken as the average performance in the three blocks of training illustrated in Fig. 1A. Motor performance for the two groups is shown in Fig. 1B and data are presented in Table 1. The likelihood ratio tests on the mixed effect model for long-term motor changes in performance showed neither a main effect of GROUP (Chi^2^_(1)_ = 0.007, p = 0.93) nor a GROUP × TIME interaction (Chi^2^_(3)_ = 0.12, p = 0.99). There was however a significant main effect of TIME (Chi^2^_(3)_ = 69.26, p < 0.001). Post-hoc tests revealed a performance increase from ‘Day 1’ to ‘6 weeks’ (10.04 points increase, z = 9.05, p < 0.001), and a similar increase was present at the ‘Retention 1’ test 8 days after the final training session (10.7 points increase, z = 9.9, p < 0.001). There was no difference in motor performance from the 6-week test to the ‘Retention 1’ test 8 days later (z = 0.84, p = 1). Motor performance 14 months after the intervention was still higher compared to ‘Baseline’ (6.83 points increase, z = 5.4, p < 0.001), but there was a decrease in performance when compared to performance at Retention test 1 8 days after the last training session (z = − 2.965, p = 0.018). Please note that model estimates for ‘Retention 2’ are based on fewer data points. Raw values for the 14 participants tested on ‘Day 1’-task level at all time-points incl. Retention 2 are shown in Table 2.

### Long-term changes in motor performance at the ‘End’-task level

After 6 weeks of training and at the delayed retention tests, motor performance was also assessed at an advanced task level for half of the participants. The task difficulty corresponded to the level that the first batch of participants in the progressive group reached during the 6 weeks of training and is hence referred to as ‘End task level’. Figure 1C shows motor performance at the ‘End task level’ for the 12 participants tested on this level, along with group means. The statistical model showed a significant GROUP × TIME interaction (Chi^2^(2) = 11.287, p = 0.0035). Post-hoc testing of relevant comparisons showed that the PT group performed better at the ‘End task level’ after 6 weeks of training (z = 4.51, p < 0.001) and 8 days later at Retention test 1 (z = 5.27, p < 0.001). Interestingly, motor performance at the End task level dropped in the PT group between retention test 1 and 2 14 months later (5.76 points decrease, z = 5.872, p < 0.001) where it was not different to that of the NPT group (z = 0.759, p = 1). Data is presented in Table 1 and examples of EMG activity during the first and last day of training are illustrated in Fig. 1D. These results demonstrate that increasing task difficulty for the PT group during the 6 weeks of training allowed them to perform significantly better at a high level of task difficulty compared to the NPT group. Furthermore, this improved motor performance persisted through 8 days but not 14 months of detraining. Please note that the model-estimate for Retention test 2 is estimated from five participants in each group (n = 10). Accordingly, raw values for the ten participants tested at all time points are presented in Table 2.

### Electrophysiological measures of corticospinal plasticity

**Figure 2:**
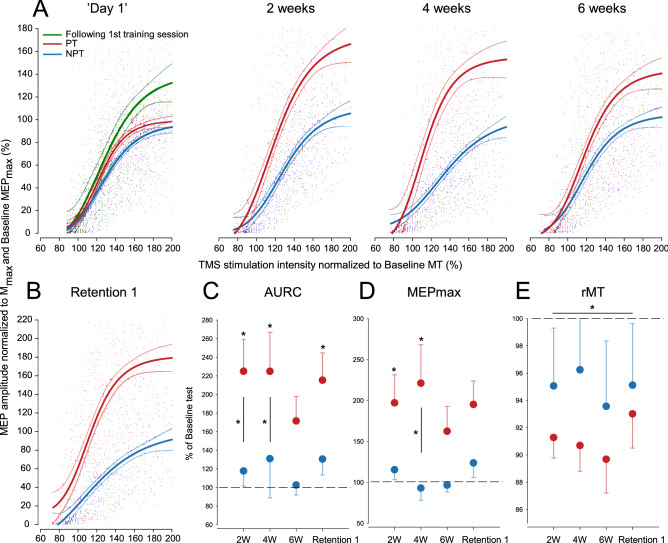
Electrophysiological results for the progressive (red) and non-progressive (blue) training group. (**A**) Pooled recruitment curves for both the PT Group (n = 12) and the NPT Group (n = 12 before and after the first day of training, as well as after 2, 4 and 6 weeks of motor practice. Dots represent motor evoked potential amplitudes normalized to M_max_ and then to baseline MEP_max_. Green dots and curve fit (left) illustrate measurements obtained immediately after the first training session. Groups are merged for this fit because the protocol for the first training session was identical for all participants. Plots represent global fits to the complete dataset, and dotted lines represent confidence bands. No statistical tests were performed based on the presented global fits, which primarily serve to illustrate the data set. (**B**) Global recruitment curves obtained at Retention test 1 following 8 days of detraining. (**C**) Group mean area under recruitment curve (AURC) normalized to individual baseline values (represented by the dashed line). Asterisks denote a significant difference from ‘Baseline’ and asterisks next to vertical black lines denote a difference between the two groups. (**D**) Group mean MEP_max_ values normalized to individual baseline value (represented by the dashed line). Asterisks denote a significant difference from ‘Baseline’ and asterisks next to vertical black lines denote a difference between the two groups. (**E**) Group mean resting Motor Threshold (rMT) normalized to individual baseline rMT (represented by the dashed line). The asterisk denotes a significant difference from ‘Baseline’ across the two groups for all time points. For (**C**–**E**) error bars represent s.e.m.

The individual TMS recruitment curves were obtained before and after the first day of training as well as after 2, 4 and 6 weeks of training and at the two retention tests. The curve fitting procedure resulted in a mean r^2^ of 0.72. In addition to TMS recruitment curves, measurements were also obtained for resting motor threshold (rMT) and maximal compound muscle potential elicited by peripheral nerve stimulation (M_max_) in each session. All motor evoked potential (MEP) amplitudes were normalized to the corresponding M_max_. There were no differences in M_max_ between groups or across time. All electrophysiological data are presented in Table 3. Additionally, recruitment curve parameter estimates were normalized to individual ‘Baseline’ values in order to investigate the relative changes in each parameter. Data normalized to individual ‘Baseline’ values are presented in Fig. 2. For illustrative purposes, all normalized recruitment curve data for all participants in the PT and NPT groups are plotted in Fig. 2A,B. The depicted curves represent global fits for all participants in the two groups in each test. No statistical comparisons were performed on the global fits.

### Changes in electrophysiological measures following the first training session

#### Area under recruitment curve

The mixed effects model for the area under the recruitment curve AURC revealed no significant interaction between GROUP and TIME (Chi^2^(1) = 2.14, p = 0.14) and no main effect of GROUP (Chi^2^(1) = 2.05, p = 0.15). There was a significant effect of TIME (Chi^2^(1) = 11.28, p < 0.001), meaning that AURC increased following the first day, i.e. after 28 min of training.

#### Maximal MEP amplitude

The model comparison for maximal MEP amplitudes MEP_max_ revealed no significant interaction between GROUP and TIME (Chi^2^(1) = 1.73, p = 0.19) and no main effect of GROUP (Chi^2^(1) = 1.67, p = 0.2). There was however a significant effect of TIME (Chi^2^(1) = 10.28, p = 0.001), indicating that MEP_max_ increased following the first day of training.

### Long-term changes in electrophysiological measures

We observed no significant effects of GROUP or TIME, and no interaction for the slope or the I50 parameter of the recruitment curves (p > 0.15). Consequently, the following results section focuses on the AURC, MEP_max_, and rMT parameters.

#### Area under recruitment curve

The mixed effects model of AURC with GROUP × TIME as fixed effects and PARTICIPANT as a random intercept effect showed a significant GROUP × TIME interaction when tested against the model without the interaction term (Chi^2^(4) = 10.11, p = 0.039). Post hoc tests of differences in AURC compared to ‘Baseline’ (eight comparisons) showed an increase in AURC after 2 weeks (124.9 ± 34.3%, z = 3.84, p = 0.001), and AURC remained elevated at 4 weeks (124.8 ± 42.2%, z = 3.47, p = 0.004) and at the Retention test 1 (115.2 ± 29.5, z = 3.7, p = 0.002) for the PT group. The 71.5 ± 26.4% increase observed in AURC after 6 weeks of training in the PT group did not reach statistical significance when correcting for multiple comparisons (z = 2.3, p = 0.17). There were no significant long-term changes in AURC for the NPT group (all p = 1). Post hoc tests for differences between groups for the $ time points (four comparisons) showed a higher AURC for the PT group following 2 (z = 2.84, p = 0.018) and 4 weeks of training (z = 3.49, p = 0.002). The difference between groups did not reach statistical significance after 6 weeks of training and at Retention test 1 (p = 0.43 and p = 0.086, respectively). The development in AURC for the two groups can be seen in Fig. 2C.

#### Maximal MEP amplitude

For MEP_max_ the analysis revealed a significant GROUP × TIME interaction (Chi^2^(4) = 9.65, p = 0.047). Post hoc Tukeys tests were conducted to compare the groups (four comparisons) and the effect of time within each group (eight comparisons). The PT group showed a significant increase in MEP_max_ following the first 2 weeks of training (195.4 ± 35.9% of ‘Baseline’; z = 2.95, p = 0.026). After 4 weeks of training, MEP_max_ was increased to 219.40 ± 48.7% of ‘Baseline’, which was also significantly higher than ‘Baseline’ (z = 3, p = 0.016). After 6 weeks, MEP_max_ was no longer significantly higher than ‘Baseline’ (160.76 ± 32.0% increase, z = 1.58, p = 0.91). At the retention test 8 days later, MEP_max_ increased again to 193.5 ± 30.2% of ‘Baseline’, which again was significantly higher than the at ‘Baseline’ test (z = 3.13, p = 0.014). The NPT group showed no significant changes in MEP_max_ at any time point (p = 1). Although the PT group generally expressed larger relative increases in MEP_max_ values after 2, 4 and 6 weeks of motor training as well as Retention test 1, a statistically significant difference was only observed following 4 weeks of practice (z = 3.57, p = 0.001, other p > 0.14) when considering between groups post hoc comparisons. Data for both groups normalized to individual ‘Baseline’ values are presented in Fig. 2D.

#### Resting motor threshold

Analysis of the relative changes in resting motor threshold (rMT) based on normalized data also revealed a main effect of TIME (Chi^2^(4) = 22.35, p < 0.001), but no main effect of GROUP (Chi^2^(1) = 0.6, p = 0.38) or interaction between GROUP and TIME (Chi^2^(4) = 3.13, p = 0.54). Post hoc comparisons for the main effect of TIME (four comparisons) showed a relative reduction in rMT by ~ 6% or more at all time points compared to ‘Baseline’, (z > 3.3, p < 0.004) Group mean values for rMT are presented in Table 3 and data normalized to ‘Baseline’ are illustrated in Fig. 2D.

#### Correlation analysis

Pearson product moment correlation analysis revealed a significant association between changes in MEP_max_ and end-level task performance at the 6-week test (r^2^ = 0.48, p = 0.018). In addition, a strong tendency towards a negative association between changes in rMT and relative changes in performance from Day1 to 6 weeks on the ‘Day 1’ task level was observed (r^2^ = 0.14 and p = 0.057). No correlation was present between either MEP_max_ at 6 weeks and motor performance at ‘Day 1-level’ after 6 weeks of training (p = 0.73).

## Discussion

Our study demonstrates for the first time that an individually adjusted, progressive training protocol involving 6 weeks of visuomotor skill acquisition results in significantly better motor performance compared to a non-progressive skill learning protocol. Progressive training was also accompanied by pronounced increases in corticospinal excitability suggesting that continuous challenge during motor training drives corticospinal plasticity. Thus, this study confirms previous findings in non-human primates indicating that learning, rather than execution, drives long-term corticospinal plasticity^27^. These findings also provide a plausible mechanistic background for increased cortical representational and/or CSE observed in expert musicians and athletes. This underlines the importance of continuously challenging motor performance for driving plastic changes and ensuring maximal performance gains. This is relevant not only for musicians and athletes but also as a plasticity inducing tool to improve the outcomes of rehabilitation in stroke survivors.

### Progressive practice leads to superior performance

In the present study, we adapted a task previously used over a similar timespan^11^. This task enables individually adjusted requirements for *motor acuity* (i.e. the ability to move faster and more accurately^36^), while being entertaining enough to engage the participant when training on a basic level of task difficulty. The task entails a partly unpredictable environment with no fixed sequences. This differentiates it from the majority of tasks previously used in long-term studies as discussed below.

The current results provide proof-of-concept that long-term (6 weeks) of progressive practice leads to better performance on a task level with high demands for accuracy and speed compared to non-progressive practice. The present results are in line with our previous findings that progressive practice facilitates skill acquisition for both the trained and untrained hand after 4 days and 6 weeks of training, respectively^4,11^. While the small number of participants tested on the high level of task difficulty (n = 12, 6 in each group) calls for a cautious interpretation, the difference between participants depicted in Fig. 1C with only one observation overlapping between groups after 6 weeks and no overlapping data at ‘Retention 1’ substantiate the claim of superior learning in the PT group. While the PT group outperformed the NPT group at a difficult task level, testing at ‘Day 1’ task level revealed similar improvements in performance between the two groups. This was observed even though the NPT had trained at this specific task level for 6 weeks. These observations suggest that progressive motor training, with greater demands for speed, attention, visual processing and precise motor output, can improve capabilities at lower task levels to a similar extent as the improvements observed with task-specific practice. As we did not systematically investigate the number of little finger abduction and adduction movements, the changes in EMG pattern and amplitude, or the active time during each training bout, we cannot definitively pinpoint the effect of progressive training to kinetics, kinematics or task exposure. It is however noteworthy that differences between groups were still present after 8 days of detraining. These results suggest persistent differences in the learning effects and support the conclusions above.

Whereas performance measures after 8 days of detraining mirrored those observed after 6 weeks of practice, a long-term retention test conducted after 14 months demonstrated that between-group differences in motor performance were no longer present. Here, both groups showed retention of skill when tested on the ‘Day 1’ task , but the benefits of progressive practice on ‘End level’ performance were no longer present. This emphasizes the necessity of continued training at a high task level in order to maintain skill mastery.

### The motor learning model

High fidelity, individual adjustments of task difficulty are a pivotal part of the deliberate practice necessary when training for skill mastery in elite sports^34^. The progressive training model provides a readily implementable method for studying the development of expertise in laboratory settings in search of determinants contributing to skill development towards expertise. Furthermore, motor learning is frequently studied by employing models of sequential learning and motor adaptation (see e.g.^37^) or categorized as either model-free or model-based learning based on the dependence on error versus reward-contingent processes^38^. However, most real life skills contain both model-based and model-free elements and require learning of motor sequences as well as the capacity to adapt to new sensorimotor environments^39^. In the present study, the participants practiced a visuomotor task (breakout), in which performance relies on accurate visuomotor transformation and fast, accurate movements. Hence, the task has a prominent motor component and improved performance is contingent on improved acuity, which differentiates it from the sequential tasks deployed in most long-term motor learning experiments (e.g.^40,41^). This is likely to be reflected in the neural correlates as discussed below.

### Initial within-session increases in corticospinal excitability

As expected, we found a left and upwards shift of the recruitment curve after the first motor practice session i.e. 30 min of visuomotor skill learning. This demonstrates that motor practice at the ‘Day 1’ task level was accompanied by an acute increase in CSE. Facilitation of TMS MEPs accompanying motor learning is supported by ample existing evidence (e.g.^4–6,42,43^). It further supports previous findings of acutely increased CSE with practice of both visuomotor^6^, ballistic^43^, and sequential^44^ motor tasks. The increased response to magnetic stimulation likely reflects processes associated with motor learning when skill automaticity is low, since the fourth^4^ and fifth^45^ day of motor practice has been found not to be associated with acute increases in CSE. It is likely that within-session changes mark top-down processes such as attention and motivation leading to successful learning, but it is yet to be investigated how such transient changes in CSE relate to the long-lasting plastic changes observed in the present study.

### Long-term progressive practice leads to increased global measures of corticospinal plasticity

We found that progressive visuomotor practice was accompanied by substantial and long-lasting increases in CSE assessed as AURC. This increase was evident as a left and upwards shift of the recruitment curve i.e. changes in the MEP_max_ and rMT parameters (see Fig. 2). In contrast, long-term NP training was only accompanied by decreased rMT.

We argue that the increased plateau level of the recruitment curve reflects cortical processes that also enable fast and precise digit control. Direct measurements from primate M1 have revealed that activity in cortical neurons is related to speed and direction of limb movement^46^ and in humans, contractions with high demands for precision are accompanied by increased corticospinal activity as reflected in increases in MEP size^47^. Furthermore ballistic learning increases MEP size^35,43,48–50^, and increasing requirements of both speed and accuracy bring about repeated gains in CSE^4^. Recently, Raffin and Siebner reported increased cortical representational map after 8 days of training on a visuomotor task comparable to the one used in the present study^51^. Interestingly, they found that changes in measures of excitability explained approximately 20% of the variance in performance gains, which corresponds well to the results of the present study (R^2^ = 0.48 and 0.14 for the ‘End’ and ‘Day 1’ task level respectively). Based on the existing literature^4,35,51^, we argue that it takes a minimum of 5 days of visuomotor training to induce learning-related representational changes in M1 that can be captured by single-pulse TMS measures. The 6 weeks of training in the present study should consequently be adequate to induce such representational changes.

In summary, existing evidence from studies in non-human primates and humans lends credence to the hypothesis that changes in the maximal corticospinal output subserve the acquisition of skilled visuomotor performance. This relation is further supported by the current findings of related changes in the MEPmax parameter and skilled performance.

### Long-term motor training leads to decreases in rMT

Motor threshold decreased for both groups during the training period, but no significant differences were observed between groups. The rMT is suggested to reflect the excitability of the most excitable elements in the stimulated pathway^52^. Recent work combining electric field modelling with modelling of cell morphology suggests that layer 2/3 and 5 pyramidal neurons located superficially at the crown of the precentral gyrus are most sensitive to TMS^53^. Changes in inputs to these or in the downstream synaptic connections could therefore account for or contribute to the reduced rMT.

It should be noted that since both groups displayed decreased rMT and no passive control group was included, it cannot be entirely excluded that motor use, passing of time, or repeated visual stimuli contributed to this decrease. However, this is unlikely as changes in rMT do not accompany unskilled motor repetition^7^.

The observed decreases in rMT with long-term motor practice in the majority of participants therefore support the ample evidence from animal research demonstrating a pivotal role of M1 in long-term motor training^20,21,25,28,54–77^.

### Changes upstream and downstream of M1 could contribute to the increased CSE

An increase in resting MEP amplitude may reflect both top-down and bottom-up processes and could in theory reflect changes in inputs from association cortices upstream from M1 engaged in skill learning but independent of the mechanisms leading to sensorimotor refinement (see^78^ for discussion). In support of this, the majority of previous investigations have failed to demonstrate a relationship between changes MEP amplitude and increased performance (e.g.^4–6,43,45,48,49,79,80^). However, in the present study, the long-term changes in MEP_max_ were related to end-level motor performance on an individual level after 6 weeks of practice. Accordingly, we suggest that the observed changes in CSE reflect task-specific adaptations in neural ensembles and single cell activity of the trained M1 as demonstrated in rodents after skilled motor practice. However, it cannot be entirely excluded that the present results reflect changes in other cortical areas than M1.

The experimental paradigm of the present study also does not permit us to exclude the possibility of contributions to the observed increase in CSE from neural structures downstream of M1. Synaptic connections between descending pyramidal cells and their spinal targets are malleable^81,82^ and are a likely substrate for activity-dependent modulation^83^. It is likely that plastic changes on a spinal level contribute to reshaping motor output during skill learning (see^84^ for review). Previous findings that short-term ballistic learning potentiates the response to electrical stimulation at the level of the brainstem support the notion that spinal adaptations may contribute to the observed changes in MEP amplitude on the first day of training^85^. However, training on a visuomotor tracking task similar to the dynamic visuomotor task used in this study does not increase the size of brainstem evoked motor potentials^85^. This suggests that the observed changes in CSE following the first day of practice are likely cortical in origin.

Due to methodological challenges, measures of subcortical plasticity such as changes in the response to cervicomedullary stimulation were not included in the present study. The position and impedance of the stimulation electrodes on the mastoids are difficult to reproduce throughout 6 weeks of measurements. Furthermore, whereas substantial discomfort would be associated with mapping the full input–output relationship in resting hand muscle, current would spread to C7/C8 cervical motoneurons at intensities below those needed to trigger a maximal compound potential^86^. Also, we did not asses changes in excitability of the motoneurons or spinal reflexes using e.g. F-waves and H-reflexes. H-reflexes are not readily elicited in ADM and comparisons between days are complicated by the inability to elicit stable reflexes on the ascending part of the H-reflex recruitment curve while an M-wave is present for stimulus control^87^. In contrast, F-waves are readily obtained from intrinsic hand muscles and have previously been obtained to estimate changes in intrinsic excitability of the motoneuronal pool (see e.g.^88^). However, the between-day reproducibility of the read-outs i.e. chrono-dispersion, persistence and amplitude^89^ as well as the interpretation of changes in these are associated with some controversy^90^. Future investigations into neural underpinnings of the behavioural effects of long-term progressive training should include measures of both intracortical and subcortical excitability to further elucidate central nervous correlates of skill learning.

### Perspectives for neurorehabilitation

Our observations have direct clinical applicability. The overarching aim of sensorimotor rehabilitation following central nervous lesions is to improve motor functions and recover independent living. This, in turn, depends on the underlying neuroplastic changes. The few daily repetitions reported for neurological patients during upper limb training (32 for stroke^91^, 7 for paraplegic and 42 after tetraplegic spinal cord injured^92^) are not likely to drive functional changes through use-dependent mechanisms. To achieve extensive central nervous reorganization comparable to those reported in laboratory animals following a > tenfold number of repetitions (e.g.^27,28^), additional measures are likely needed. Despite substantial attention to deliberate practice both within and outside of the scientific community, an emphasis on deliberate training for expertise has not gained much influence in neurorehabilitation. The finding that a continuously challenging training regime boosts corticospinal plasticity and elicits additional behavioural effects with a similar volume of training has extensive implications for rehabilitation. Based on the present behavioural results and underlying changes in corticospinal transmission, we suggest carefully monitored progressive training protocols as routine choice in neurological rehabilitation.

## Conclusions

We demonstrate that continuously challenging the individual participant by progressively increasing task difficulty during long-term motor practice enhances motor learning and optimizes performance in a complex discrete motor task requiring highly accurate and rapid movements and with high demands for visual processing and movement prediction. Progressive long-term training not only enhanced learning, but also prolonged increases in corticospinal plasticity without increasing the volume of training. In conclusion, the results suggest that motor learning paradigms should be structured with the aim of continuously challenging the individual. This is critical in order to promote the neuroplasticity that underlies learning and therefore to improve the behavioural outcome of training for neurorehabilitation following acquired brain injury and for athletes who engage in training.

## Materials and methods

We obtained measures of motor performance and applied transcranial magnetic stimulation (TMS) and peripheral nerve stimulation to assess the effects of two different training protocols on motor skill learning and changes in corticospinal excitability. Two groups of participants engaged in 6 weeks of visuomotor training with either maintained (NPT) or progressively increased task difficulty (PT). Training consisted of a visuomotor task developed for this study. The effect of training on corticospinal excitability was assessed using TMS by comparing recruitment curve parameters before training and after 2, 4 and 6 weeks of motor practice. Delayed retention of motor performance and CSE were assessed at Retention test 1 and 2, 8 days and 14 months after the 6-week training intervention respectively, to evaluate the long-term effects of motor practice.

### Participants

Twenty-four adult men aged 21–29 years at the time of enrolment (24 ± 4, mean ± s.d.) were randomly allocated into two different training groups. All participants had a moderate to high level of daily physical activity and had no known medical condition that could interfere with motor skill learning of hand movements. Participants were matched in pairs based on initial visuomotor performance i.e. during the first training session. Each member of the pair was randomly assigned to one of the two groups to ensure comparable ‘Day 1’ performance in the two groups. For details of the performance test, see below. Participants were instructed not to engage in physical training of any kind prior to testing sessions and to eat, sleep and drink similarly on all days of testing. For each subject, all tests were conducted at the same time of the day to minimize intra-individual day-to-day differences in motor cortical excitability^52^. Twenty-three participants were right handed according to the Edinburgh Handedness Inventory^93^ and one had no hand preference.

Written informed consent was obtained from all participants prior to their participation in the study. The experiments were approved by the local ethics committee of the capital region of Denmark (KF01-131/03) and all experimental procedures were carried out in accordance with the Helsinki Declaration (1964).

### Design

The two groups of participants engaged in 18 training sessions with their right (dominant) hand over a period of 6 weeks. Training sessions were held three times per week and, when possible, separated by 48 h. Each training session consisted of seven 4-min bouts of activity interspaced with 2-min rest periods. Electrophysiological testing was repeated at 2-week intervals, and behavioural performance was evaluated during the first training session and assessed after 6 weeks of motor training. Eight days after the end of the 6-week training period, all participants were subjected to a retention test i.e. a delayed test of motor skill retention and CSE. Fourteen months after the training period, all available participants (n = 14) were subjected to a second delayed test of motor skill retention.

**Figure 3:**
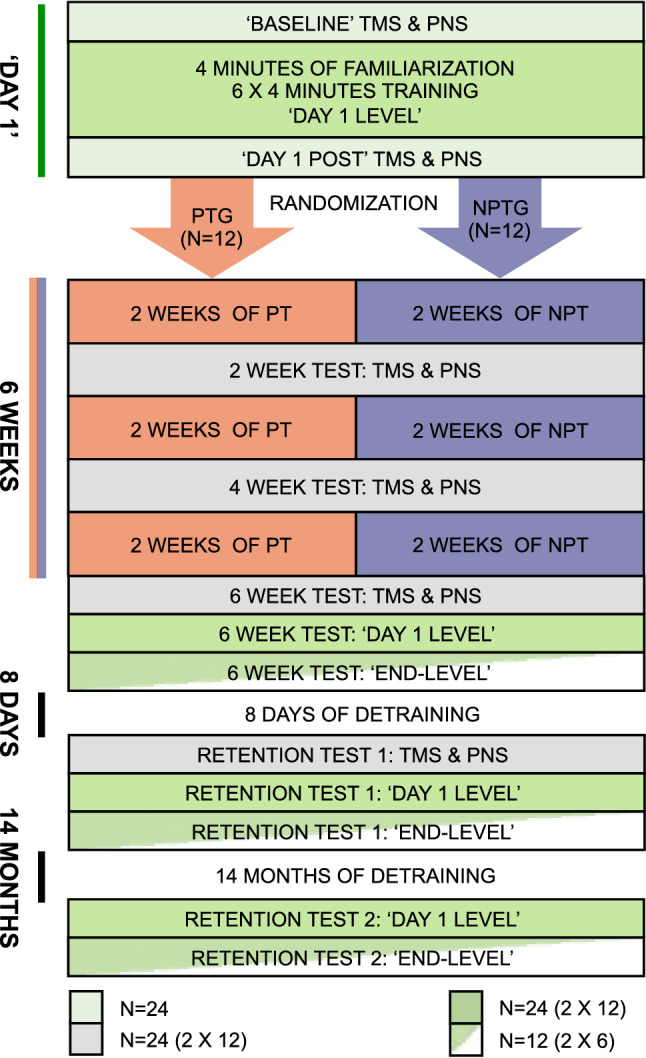
General design of the study. Flow chart illustrating the design of the study. Please note that the study was conducted in two ‘batches’ of 12 participants each. The velocity and paddle size of the ‘End’ task level was established based the attained performance progression of the six participants engage in progressive skill training from the first ‘batch’, whereas the group-comparison of performance on the ‘End’ task level was based on six participants from each group of the last ‘batch’(N = 12).

At least 3 days prior to the ‘Day 1’ session all participants were familiarized to transcranial magnetic stimulation. During the ‘Day 1’ session, TMS recruitment curves were obtained both before training and again immediately after the post training M_max_, which is described below. The electrophysiological measurements obtained pre-training on Day 1 were used for long-term comparisons and are referred to as ‘Baseline’. After 2, 4, 6 weeks of training and in the delayed retention tests after 8 days and the detraining period, only pre-training electrophysiological measurements were obtained. At the 6-week test and in the long-term retention tests 8 days and 14 months after the intervention, electrophysiological measurements were obtained initially and motor performance was tested subsequently. The testing and training were conducted in 2 “batches” due to limited laboratory capacity i.e. 12 participants (six in each group) underwent testing and training procedures in parallel. Testing of a 2nd ‘batch’ also consisting of 12 participants (six in each group) started following the 6 weeks testing of the first “batch”. An overview of the long-term study design is presented in Fig. 3.

It should be noted that the results from the first six participants in the PT group were used to estimate the attained ‘end-level’ motor performance. It was therefore only possible to test the remaining half of the participants from each group at this task level. This was done after 6 weeks of training and at the retention tests.

The first training session took place at the ‘Day 1’ session and was identical for the two groups. After the first training, one group continued training at the ‘Day 1’ level (non-progressive training group, NPT), while the other group trained with task-difficulty that was progressively adjusted to correspond to their skill level in the motor task (progressive training group, PT). No adjustments to the level of difficulty were made for NPT group. For details on the progression, see below. Only the 2nd batch of participants was tested at the ‘End Level’ of task difficulty (i.e. ‘End level’) at the 6 W and retention tests (see Fig. 3).

### The Visuomotor task

**Figure 4:**
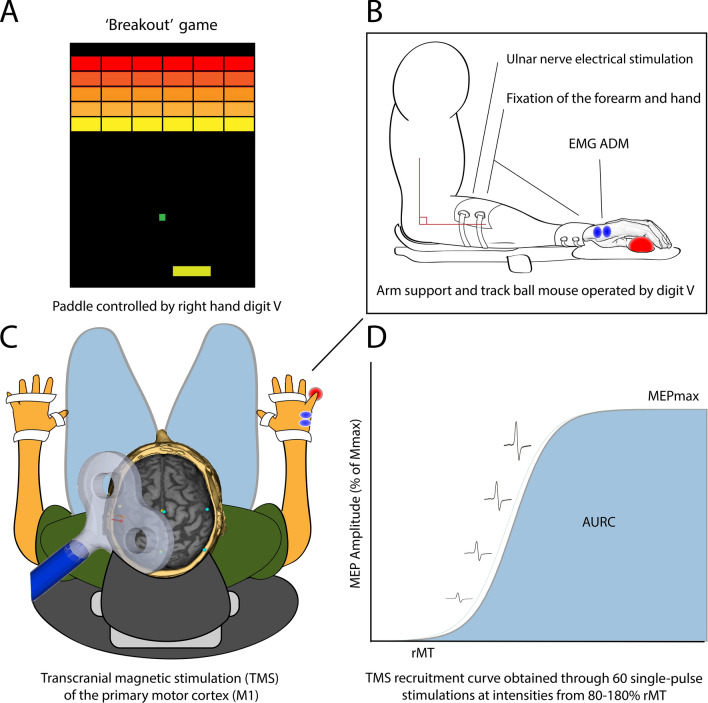
Behavioural task and experimental Setup. The visuomotor training task consisted of a game called “BreakOut”, a spin-off from a classical arcade game. (**A**) A screen shot from the game shows a random in-game situation. (**B**) The paddle at the bottom of the screen was moved by rolling the trackball to the right or left by abducting or adducting the fifth digit. Hand position during motor practice. (**C**) Both of the subject’s arms and hands were strapped during electrophysiological measurements in order maintain stable hand and arm position. (**D**) Representative Motor Evoked Potentials (MEPs) normalized to M_max_ at different transcranial magnetic stimulation (TMS) intensities (normalized to resting motor threshold (rMT) for one subject.

The motor task consisted of a visuomotor game called “BreakOut”, a spin-off from a classic arcade game (see Fig. 4). Participants were able to move a small paddle presented at the bottom of the screen using a trackball, which was controlled abduction or adduction of the fifth digit. The paddle was moved by rolling the trackball in order to make a ball bounce between the paddle and a level-dependent collection of bricks (80–120) with the purpose of eliminating bricks. If the paddle was not positioned correctly, the ball would move past the brick and be lost. Then, a new ball would appear at the top of the screen and the game would continue. Losing three balls caused the game to start over with the original number of bricks restored. Participants were instructed to ‘eliminate as many bricks as possible without losing the ball’. The speed of the ball, size of the paddle and number of bricks were adjusted in order to modify the difficulty of the game in accordance with a previously determined progression order^11^. Either an increase in speed or a decrease in paddle width would increase the demand for motor acuity. Acutely, this caused a decrease in the number of bricks eliminated per ball and therefore the duration over which each ball was in play. With practice, more bricks were eliminated per ball and the duration of continuous play increased. In order to progress from one game level to the next, the screen had to be cleared three times during the same training, continuously increasing the difficulty on an individual level for each participant in the progressive group.

In each training session the participant started out by training at the level of task difficulty that was reached during the previous training. During performance testing, the total number of residual bricks and lost balls were recorded for 3 (‘Day 1’) or 2 (at 6 weeks and retention tests) of the training blocks for later analysis. Participants received standardized information about the game and the performance score and were asked to do their best at all times. The first 4 min of training on Day 1 served as a familiarization procedure and performance measures from these 4 min were not included in the data analysis.

During training (and testing) the participant was seated in a comfortable chair with both hands on a panel placed on top of a table. The right hand and forearm were secured with Velcro^TM^ straps to maintain the hand position during training. The forearm was kept flat on the panel by two straps; one distal to the elbow joint and the other approximately 2 cm proximal to the wrist. The hand was held in a pronated position by two straps, one distal to the wrist and the other crossing the back of the hand. The thumb and first three fingers were also fixed to the panel by two straps. The trackball was built into the supporting panel and positioned below the fifth digit. The participants manipulated the trackball and thereby the position of the game-paddle by abducting and adducting the finger. The experimental setup is illustrated in Fig. 4. The participant was positioned with the shoulder slightly flexed and abducted and with elbow joint flexed to approximately 90°. During electrophysiological measurements, the left hand was placed in a relaxed position similar to the right arm.

### Recording and stimulation procedures

Electromyographic (EMG) recordings from the ADM muscle were obtained with bipolar surface EMG electrodes (0.5 cm diameter of electrodes; 2 cm distance between electrodes; Blue Sensor, Ambu Inc.,USA) over the belly of the muscles. The EMG signals were amplified (2000×), using NeuroLog EMG amplifiers (Digitimer Ltd., UK), band-pass filtered (5 Hz–1 kHz) sampled at 2 kHz, and stored on a PC for off-line analysis (CED 1401+ with Signal 3.09 software, Cambridge Electronic Design Ltd., UK). EMG activity during training was recorded with Spike 2 (CED, Cambridge UK) and stored for later off-line analysis.

Magnetic stimuli were delivered to the contralateral (left) hemisphere primary motor cortex (M1) by a Magstim Rapid^2^ stimulator (Magstim Company Ltd., Whitland, UK) via a custom made 90 mm figure-of-eight coil (batwing design, Magstim Company Ltd., Whitland, UK) with the capability to deliver a magnetic field of 2 T. The optimal coil position (hotspot) for eliciting motor evoked potentials (MEPs) in the ADM muscle was established through a standardized stimulation procedure with high spatial resolution covering the primary motor cortex (M1), i.e. a mapping procedure, at each test. During assessment of the resting motor threshold (rMT) and during generation of the recruitment curves the coil was placed with the centre oriented parallel to the scalp over the hot-spot of the ADM representation with the handle of the coil pointing backward at an angle of 45° to the sagittal and horizontal axis. TMS recruitment curves were obtained by delivering 60 single pulse stimuli in a random sequence with an inter-stimulus interval of 3 s and stimulus intensities ranging from 0.8 to 1.8 MT^94^. The rMT was defined as the minimum intensity required to elicit a peak-to-peak MEP amplitude larger than 2 × s.d. of average background activity (i.e. noise) in three out of five trials (always below 50 μV). All TMS measurements were obtained while the participant was at rest. Trials in which any background activity larger than 2 × s.d. was observed were discarded. A maximum of five trials were discarded from each recruitment curve, which consequently was modelled based on at least 55 MEP amplitudes. During all experiments involving TMS, frameless stereotaxy (Brainsight 2, Rogue Research, Montreal, Canada) was used to identify the coordinates of the M1 hotspot and to monitor the position of the coil relative to the participants’ head.

Before generation of recruitment curves at each test, maximal compound muscle action potentials of ADM (maximal M-waves, M_max_) were elicited by bipolar electrical stimulation of the ulnar nerve (Digitimer 7A constant current stimulator, Digitimer Ltd., UK). The intensity of stimulation was increased from a subliminal level until there was no further increase in the peak-to-peak amplitude of the M-wave with increasing intensity. The purpose of this procedure was to normalize the MEP data obtained on each test day to the corresponding M_max._ This allowed comparison across different test sessions. Assessors were not blinded to the group allocation.

### Data analysis and statistics

Visuomotor performance was computed as the average number of bricks shot down by each ball corrected by a factor 1.*n* with *n* being the number of screens/rounds cleared without losing the ball within each 4 min block, i.e. a bonus for completing trials successfully. Each 4-min block of motor training or test of motor performance thereby resulted in a cumulated score. This correction accounts for the fact that the elimination of the last few blocks is associated with greater difficulty and time use. During the first day of practice (‘Day 1’) the performance scores from the first 4, middle 4 and last 4 min were used to depict learning during the first session (see Fig. 1A). The average of these 12 (3 × 4) min of training was used as the ‘Day 1’ score in long-term performance comparisons. For the remaining tests, performance measures were obtained from two blocks of 4 min of testing on the ‘Day 1’ task level. The reduced sampling time was chosen as it adequately represented the performance level of the participants and with the intention of minimizing the influence on later electrophysiological recordings (i.e. on retention tests). For the 12 participants tested in the 2nd test-round (see Fig. 3) the reduced test time also reduced potential anterograde interference of the testing on the ‘End’-task levels.

MEP amplitudes were normalized to M_max_ (recorded just prior to TMS testing on that day) to allow comparison between test days, and stimulation intensity was normalized to ‘Baseline’ rMT. The MEP amplitudes obtained *after* the first training session were normalized to M_max_ likewise obtained after the training. The recruitment curves were constructed by modelling the relationship between stimulus intensity and MEP peak-to-peak magnitude with a Boltzmann-like sigmoid equation previously described^95,96^. The equation relating the magnitude of the MEP to the stimulus intensity (*I*) is:

$$ {\text{MEP}}\left( {\text{I}} \right) = {\text{MEP}}_{\min } \frac{{{\text{MEP}}_{\max } - {\text{MEP}}_{\min } }}{{1 + {\text{e}}^{{\frac{{{\text{I}}_{50} - {\text{I}}}}{{\text{S}}}}} }} $$

where MEP_min_ is the amplitude of background noise, MEP_max_ is the maximum plateau value, I_50_ is the stimulus intensity at the inflection point where a MEP amplitude of 50% of MEP max is obtained and S is the slope at the inflection point. The inverse of the slope parameter (1/S) is directly proportional to the maximal steepness of the function. Thus, the MEP recruitment curve is described by the motor threshold (rMT), the maximum elicited response (MEP_max_), and the transition between them (S, I_50_) in relation to stimulus intensity (I)^94^. The parameters were estimated by fitting this equation to the stimulus–response data with a standard Marquardt–Levenberg non-linear least squares algorithm (Matlab curve fitting toolbox). Based on the parameter estimates, the area under recruitment curve (AURC) between 80 and 180% of rMT was calculated for each individual at each test as a global measure of CSE^97^.

All statistical analyses were carried out in R (version 3.4.1, R Core Team, 2017). A linear mixed effect analyses was computed using *lme4*^98^ and multiple comparisons computed using *multcomp*^99^. The linear mixed models include the dependent variable modelled against GROUP and TIME (with and without interaction term) as fixed effects and PARTICIPANT as a random effect with random intercept. In order to investigate the relative changes in corticospinal excitability and to ensure that each participant would have the same influence on the statistical analysis, the stimulus response curve parameters described above including rMT were furthermore normalized to individual ‘Baseline’ values. All values are reported as mean ± standard error of the mean (SEM) unless stated otherwise. Homoscedasticity and normality were assessed through visual inspection of the residual and quantile–quantile plots and data was log-transformed if any obvious deviations were observed. p-values were obtained by likelihood ratio tests of the full model against the model without the effect in question. In all tests, statistical significance was accepted at the p < 0.05 level and all a priori specified post hoc pairwise comparisons were Bonferroni corrected. Three post hoc comparisons were conducted to investigate the changes in performance for all 24 participants during the first training and six were conducted to assess long-term effects of motor training on ‘Day 1’ task level performance. Seven comparisons were carried out to test for differences at 6 weeks and the two retention tests on the task ‘End-level’. To investigate within-group long-term changes in CSE compared to ‘Day 1’, eight comparisons were conducted and additional four to investigate differences between groups.

## Data Availability

The datasets generated and analysed during the current study are available from the corresponding author on reasonable request.
